# Typhoid Fever, as It Prevailed in Germantown, Tennessee, during the Fall of 1847

**Published:** 1848-02

**Authors:** R. L. Scruggs

**Affiliations:** Communicated in a letter to Mr. J. L. Adkins, Student of Medicine in Jefferson Medical College


### MEDICAL EXAMINER,

AND

### RECORD OF MEDICAL SCIENCE.

NEW SERIES.—No. XXXVIII.—FEBRUARY, 1848.

---

### ORIGINAL COMMUNICATIONS.

---

*Typhoid Fever, as it prevailed in Germantown, Tennessee, during the fall of* 1847. By R. L. SCRUGGS, M. D. Communicated in a letter to Mr. J. L. ADKINS, Student of Medicine in Jefferson Medical College.

This disease made its appearance in our vicinity in the latter part of August last. The first case that I was called to see, was the wife of a neighbouring physician; and it is probable that here I should have failed to recognize the disease, but from the fact that two years ago I assisted in the treatment of two similar cases at the same plantation. This circumstance, with the violence of the symptoms present, caused me to make a more careful examination than probably I should otherwise have done, which, fortunately for me, resulted in a correct diagnosis. For, although there is some difficulty in diagnosticating between this disease at its commencement, and bilious remitting fever, yet, when the attention is called to it, the difference appears to me to be sufficiently manifest; the pulse is peculiar, and, I think, pathognomonic of the disease.

Up to this time, I have treated and assisted in the treatment of twenty-two cases: of these, seven occurred at one plantation, eight at another, three at another, and four other places furnished one case each. Out of these, only one case terminated fatally, which result was owing, I think, to the patient's refusing obstinately to submit to treatment. This patient died on the thirty-first day of her confinement. She miscarried during this time; I think about the fifteenth day of her illness. She ate ice, and drank freely of iced water from a week after she was taken sick until within a short time before she died, in opposition to my opinion, and in spite of the remonstrances of her husband.

The premonitory symptoms usually were loss of appetite, languor, debility and indisposition to bodily or mental exercise. This lasted for a day or two, when they would take to their beds, with more or less fever, pain in the head, thirst, dry, hot skin, tongue red at the tip and edges, with a dirty white, or drab-coloured coat in the centre; the abdomentender to the touch, with nausea and sometimes vomiting. In all there was a tolerably intense gastro-enteritis, great prostration of muscular power, with that peculiar strike of the pulse, which is not easily described, but which, once recognized and understood, will never fail, I think, to enlighten us as to the true character of the disease.

*Etiology.*—The cause of Typhoid fever, as it occurs in our country, is extremely obscure. We have a few sporadic cases every year, and sometimes the number of cases amongst us entitles it to the name of epidemic. It is remarked, by those who have had the best opportunities of observing the course of the disease in this country, that the cases are most numerous in those years that are said to be most healthy; in other words, in those years in which we are least troubled with intermitting and remitting fevers. Thus a season generally healthy, gives us, in the months of August, September and November, the greatest number, and most violent cases, of Typhoid fever. It is not influenced by the laws which govern intermitting and remitting fevers—not under the control of bark and its preparations; thus showing that it is caused by a peculiarity of the atmosphere totally and entirely different from that which developes periodic diseases. That it is not contagious is equally clear to my mind, from the fact that we often have but one case at a plantation, although these solitary cases occur at places with a population of from thirty to eighty slaves, all of whom visit, and pay more or less attention to the sick one, during his or her illness.

*Treatment.*—Only in two cases have I deemed it prudent to take blood from the arm, and both of these patients were convalescent on the ninth day, and recovered more rapidly than any of the others. My rule is to bleed whenever the pulse will justify it. The average duration of the disease was about twenty-one days. Many patients, however, remain in a weakened and debilitated condition for several weeks after the disease is arrested. These require stimulants, judiciously administered, tonics, moderate exercise and nutritious food. Where venesection cannot be borne, I invariably commence the treatment with one thorough cupping over the whole abdomen, which I extend to the chest if there is the least evidence that the thoracic viscera participate in the inflammation ; this to be followed by a warm emollient poultice ; after which stimulating embrocations, to be followed by pepper mush poultices. It was sometimes necessary to repeat the cupping, and always necessary to continue, at least, as long as there was much evidence of active inflammation of the bowels, the stimulating frictions and pepper mush poultices. Blisters, in a large majority of cases, in the latter stages of the disease, are indispensable. They may be used on one part of the abdomen or chest, while the poultices are applied over all the other parts ; or, after they draw properly, are clipped and dressed, they may be covered with a cloth and the poultices spread over them. There was sometimes pain in the back part of the head, with a sort of stiffness and pain in the neck, which was relieved by the cups and the application of a blister to the nape of the neck. The blister here, too, seemed to relieve the patient of a certain puffiness that was observed about the eyes. We frequently used the foot-bath, particularly when the head was painful, and generally made stimulating with salt, mustard or pepper, and sometimes with all of these articles. Cold applications to the head were very seldom resorted to, because the patients objected to them. And I would here remark that I make it a rule, which I think is applicable in all cases, never to apply cold water to the head, or any other part of the body, when the sensation produced by it appears to produce unpleasant feelings to the patient.

Internally, we commenced by giving from two to five grains of calomel with ipecac: repeated every third hour, or until a free discharge from the bowels was procured. If, after the third or fourth dose was administered, the bowels were not moved, then a tea-spoonful of castor oil, and fifteen drops of spirits of turpentine, in a little elm water, was given every third or fourth hour, until this object was attained; and to promote diaphoresis, spt. nit. dulc. and vin., ipecac. every hour. After this, the calomel and ipecac. in smaller doses, was ordered to be given once or twice a day, and the bowels to be kept soluble with cremor tartar, magnesia or sulphur. Or, if the passages from the bowels were too frequent, watery, mucous, or bloody, the hydr. c. creta, tannin, pulv. g. arabic and Dovers powders, were substituted for the sub. chloride. In one instance the hemorrhage from the bowels was so copious as to make it necessary to use the acet. of lead with opium. As diaphoretics, and to keep down arterial excitement, we frequently combined with the spirit. nit. dulc. the wine of colchicum and tinc. digitalis. Also in the latter stages of the disease, the spiritus mindereri; (and when the pulse was below the natural standard,) with an excess of ammonia. We rejected the antimonials, from fear of their irritating influence upon the inflamed bowels. Mucilaginous drinks have been strictly enjoined from the commencement of the treatment.

I am perfectly satisfied of the entire inutility of quinine in the treatment of this disease. I have used it under a variety of circumstances, with old and young patients, of both sexes, in large and small doses, alone, and variously combined; and I must say, that, so far from deriving benefit from its use, I have invariably found it to do harm. I am of opinion, however, that late in the disease, or, more properly speaking, after the disease is gone, and nothing remains to be treated but debility, that then, and not till then, can quinine and the other preparations of bark be used with advantage. Under these circumstances, I have often given it, and with marked good effects—the patients gaining their wonted strength and vigour rapidly, under the use of small doses of bark or quinine, well-regulated diet, proper exercise, &c.

I will here remark, that we have treated several cases recently, resembling these under consideration, in every respect, except that they had pneumonia along with it, constituting what I should call genuine pneumonia typhoides. I merely mention this because my mind has been much perplexed recently in reading the description of typhoid pneumonia by Prof. Dickson and others. To my mind they confound the disease with what I call catarrho-rheumatic fever, even with erysipelatous inflammations, and other diseases. I can see no reason for affixing the appellation of pneumonia to a disease because it has proved fatal, and was followed by a number of cases of pneumonia, when it is not shown, either by the symptoms, or physical signs during life, or by post mortem examination, that the parenchymatous structure of the lungs was in a state of inflammation, or that those organs participated at all in the diseased action which terminated the life of the patient.

Prof. Dickson, while treating of this disease, and under the head of anomalous cases, uses this language: "Others again seemed to be taken off by the most inadequate ailments," dying, as the phrase was, "of a pain in the foot or knee—in the ankle or wrist." I would simply ask, was this pneumonia? What I understand by typhoid pneumonia is nothing more nor less than Typhoid fever with pneumonia superadded to the other lesions. The pneumonia may supervene upon the fever, or it may be simultaneous with it. Our treatment of these cases has been nearly the same as that adopted in the typhoid fevers. The only additional articles used were expectorants. The condition of the stomach and bowels forbade the use of antimony. We have, as yet, lost none of these patients.

